# Supplementary material for: The glutathione import system satisfies the Staphylococcus aureus nutrient sulfur requirement and promotes interspecies competition
Source: PLoS Genet. 2023 Jul 7;19(7):e1010834. doi: 10.1371/journal.pgen.1010834 (PMC10355420; doi:10.1371/journal.pgen.1010834)
Supplement: S1 Text — (DOCX) [file pgen.1010834.s001.docx]

**S1 Text Supporting Materials and Methods**

**Ethics Statement**

This study was conducted in meticulous accordance with the recommendations in the Guide for the Care and Use of Laboratory Animals of the National Institutes of Health. The approved protocol, PROTO201800068, was reviewed by the Animal Care and Use Committee at Michigan State University.

**Domain prediction of GisABCD-Ggt.** The USA300_FPR3757 (assembly GCF_000013465.1) reference was used to predict domain architectures for GisA (ABD21741.1), GisB (ABD21022.1) GisC (ABD20640.1), GisD (ABD22752.1), and Ggt (ABD22038.1) using MolEvolvR [1]. Protein sequences were analyzed with custom scripts using InterProScan, TMHMM, Phobius, Pfam, and PROSITE to identify domains, secondary structures, and cellular localization signatures [2-7]. Domain architectures were visualized within MolEvolvR using custom R scripts and the R package, gggenes [1].

**GisA ATPase activity assay.** ATPase activity of purified recombinant GisA was monitored using the Malachite Green Phosphate assay (Millipore-Sigma). Recombinant GisA was diluted to 1 µg per reaction. Diluted GisA, 250 µM MgCl_2_, and 400 µM ATP were incubated for 1 h at 37°C and samples were taken at 0, 15, 30, 45, and 60 min. At the indicated time points samples were flash frozen in a dry-ice ethanol bath and stored at -80°C [8]. Samples were thawed at room temperature, Malachite Green reagent was added, and P_i_ release was determined following the manufacturer’s instructions. Reactions containing ATP in the absence of GisA were used to correct absorbance measurements due to residual inorganic phosphate and non-enzymatic ATP hydrolysis. Each biological replicate used an independently purified GisA preparation.

**Murine systemic infections.** WT and *gisB*::Tn were cultured in TSB overnight at 37°C, diluted 1:100 into TSB, and cultured for 3 h at 37°C at 225 rpm shaking. Cultures were pelleted, washed with PBS, and normalized to OD_600_ equal to 0.4. Female C57BL/6J mice were retro-orbitally infected with 10^7^ CFUs and infection proceeded for 96 h after which heart, liver, and kidneys were collected and homogenized in 1 mL PBS. Organ homogenates were serially diluted and plated onto TSA. Bacterial burdens quantified as CFUs mL^-1^ were determined. Infections were performed at Michigan State University under the principles and guidelines described in the Guide for the Care and Use of Laboratory Animals [9]. Animal work was followed as approved by Michigan State University Institutional Animal Care and Use Committee (IACUC) approved protocol number 12/16-205-00.

**Supporting References**

1. Burke JT, Chen SZ, Sosinski LM, Johnston JB, Ravi J. MolEvolvR: A web-app for characterizing proteins using molecular evolution and phylogeny. 2022.

2. Krogh A, Larsson B, von Heijne G, Sonnhammer EL. Predicting transmembrane protein topology with a hidden Markov model: application to complete genomes. J Mol Biol. 2001;305(3):567-80. doi: 10.1006/jmbi.2000.4315. PubMed PMID: 11152613.

3. Kall L, Krogh A, Sonnhammer EL. Advantages of combined transmembrane topology and signal peptide prediction--the Phobius web server. Nucleic Acids Res. 2007;35(Web Server issue):W429-32. Epub 20070505. doi: 10.1093/nar/gkm256. PubMed PMID: 17483518; PubMed Central PMCID: PMCPMC1933244.

4. Sigrist CJ, Cerutti L, de Castro E, Langendijk-Genevaux PS, Bulliard V, Bairoch A, et al. PROSITE, a protein domain database for functional characterization and annotation. Nucleic Acids Res. 2010;38(Database issue):D161-6. Epub 20091025. doi: 10.1093/nar/gkp885. PubMed PMID: 19858104; PubMed Central PMCID: PMCPMC2808866.

5. Blum M, Chang HY, Chuguransky S, Grego T, Kandasaamy S, Mitchell A, et al. The InterPro protein families and domains database: 20 years on. Nucleic Acids Res. 2021;49(D1):D344-D54. doi: 10.1093/nar/gkaa977. PubMed PMID: 33156333; PubMed Central PMCID: PMCPMC7778928.

6. Mistry J, Chuguransky S, Williams L, Qureshi M, Salazar GA, Sonnhammer ELL, et al. Pfam: The protein families database in 2021. Nucleic Acids Res. 2021;49(D1):D412-D9. doi: 10.1093/nar/gkaa913. PubMed PMID: 33125078; PubMed Central PMCID: PMCPMC7779014.

7. Quevillon E, Silventoinen V, Pillai S, Harte N, Mulder N, Apweiler R, et al. InterProScan: protein domains identifier. Nucleic Acids Res. 2005;33(Web Server issue):W116-20. doi: 10.1093/nar/gki442. PubMed PMID: 15980438; PubMed Central PMCID: PMCPMC1160203.

8. Rule CS, Patrick M, Sandkvist M. Measuring In Vitro ATPase Activity for Enzymatic Characterization. J Vis Exp. 2016;(114). Epub 20160823. doi: 10.3791/54305. PubMed PMID: 27584824; PubMed Central PMCID: PMCPMC5091952.

9. Guide for the Care and Use of Laboratory Animals. The National Academies Collection: Reports funded by National Institutes of Health. 8th ed. Washington (DC)2011.
