# Supplementary material for: The glutathione import system satisfies the Staphylococcus aureus nutrient sulfur requirement and promotes interspecies competition
Source: PLoS Genet. 2023 Jul 7;19(7):e1010834. doi: 10.1371/journal.pgen.1010834 (PMC10355420; doi:10.1371/journal.pgen.1010834)
Supplement: S1 Table — (DOCX) [file pgen.1010834.s002.docx]

| **S1 Table Staphylococcus strains used in this study.** | | |
| --- | --- | --- |
| **strain** | **description** | **reference** |
| **methicillin-resistant *S. aureus*** | | |
| JE2 | Laboratory derived wild type MRSA; USA300 LAC; CC8 | [1] |
| MW2 | MRSA, USA400; CC1 | [2, 3] |
| COL | MRSA; CC8 | [4, 5] |
| SF8300 | CA-MRSA; USA300; CC8 | [6] |
| TCH1516 | MRSA; USA300; CC8 | ATCC |
| *SAUSA300_0200*::Tn | NTML NE392 *bursa aurealis* transposon (Tn) mutant, Erm^R^ *gisA* | [1] |
| *SAUSA300_0201*::Tn | NTML NE541 *bursa aurealis* Tn mutant, Erm^R^ *gisB* | [1] |
| *SAUSA300_0202*::Tn | NTML NE457 *bursa aurealis* Tn mutant, Erm^R^ *gisC* | [1] |
| *SAUSA300_0203*::Tn | NTML NE215 *bursa aurealis* Tn mutant, Erm^R^ *gisD* | [1] |
| *ggt*::Tn | NTML NE254 *bursa aurealis* Tn mutant, Erm^R^ *ggt* | [1] |
| *gisA*::Tn | *gisA* mutant strain backcrossed into JE2 | this study |
| *gisB*::Tn | *gisB* mutant strain backcrossed into JE2 | this study |
| *gisC*::Tn | *gisC* mutant strain backcrossed into JE2 | this study |
| *gisD*::Tn | *gisD* mutant strain backcrossed into JE2 | this study |
| *ggt*::Tn | *ggt* mutant strain backcrossed into JE2 | this study |
| *∆gis* | in-frame deletion of *gisABCD*-*ggt* in JE2 | this study |
| JE2 pOS1 P*_lgt_* | JE2 harboring pOS1 P*_lgt_* empty vector | this study |
| *ggt*::Tn pOS1 P*_lgt_* | backcrossed JE2 *ggt*::Tn harboring pOS1 P*_lgt_* empty vector | this study |
| *ggt::*Tn pOS1 P*_lgt_*::*ggt* | backcrossed JE2 *ggt*::Tn harboring pOS1 P*_lgt_*::*ggt* | this study |
| *ggt*::Tn pOS1 P*_lg_*_t_::*ggt*-His | backcrossed JE2 *ggt*::Tn harboring pOS1 P*_lgt_*::*ggt* encoding a His-tag | this study |
|  |  |  |
| **clinical isolates** |  |  |
| 1055 | MRSA abscess hand cellulitis | this study |
| 1056 | MRSA abscess left arm | this study |
| 1057 | MRSA left wrist/ index finger | this study |
| 1059 | MSSA bone from the coccyx/chronic osteomyelitis | this study |
|  |  |  |
| ***Staphylococcus epidermidis* strains** | | |
| *Staphylococcus epidermidis* | strain RP62a | [7] |
| *Staphylococcus epidermidis* | clinical isolate | this study |

**Supporting References**

1. Fey PD, Endres JL, Yajjala VK, Widhelm TJ, Boissy RJ, Bose JL, et al. A genetic resource for rapid and comprehensive phenotype screening of nonessential *Staphylococcus aureus* genes. mBio. 2013;4(1):e00537-12. Epub 20130212. doi: 10.1128/mBio.00537-12. PubMed PMID: 23404398; PubMed Central PMCID: PMCPMC3573662.

2. From the Centers for Disease Control and Prevention. Four pediatric deaths from community-acquired methicillin-resistant Staphylococcus aureus--Minnesota and North Dakota, 1997-1999. JAMA. 1999;282(12):1123-5. PubMed PMID: 10501104.

3. Mashruwala AA, Guchte AV, Boyd JM. Impaired respiration elicits SrrAB-dependent programmed cell lysis and biofilm formation in *Staphylococcus aureus*. Elife. 2017;6. Epub 20170221. doi: 10.7554/eLife.23845. PubMed PMID: 28221135; PubMed Central PMCID: PMCPMC5380435.

4. Dyke KG, Jevons MP, Parker MT. Penicillinase production and intrinsic resistance to penicillins in *Staphylococcus aureus*. Lancet. 1966;1(7442):835-8. doi: 10.1016/s0140-6736(66)90182-6. PubMed PMID: 4159958.

5. Sabath LD, Wallace SJ, Gerstein DA. Suppression of intrinsic resistance to methicillin and other penicillins in *Staphylococcus aureus*. Antimicrob Agents Chemother. 1972;2(5):350-5. doi: 10.1128/AAC.2.5.350. PubMed PMID: 4494489; PubMed Central PMCID: PMCPMC444319.

6. Diep BA, Stone GG, Basuino L, Graber CJ, Miller A, des Etages SA, et al. The arginine catabolic mobile element and staphylococcal chromosomal cassette mec linkage: convergence of virulence and resistance in the USA300 clone of methicillin-resistant *Staphylococcus aureus*. J Infect Dis. 2008;197(11):1523-30. doi: 10.1086/587907. PubMed PMID: 18700257.

7. Vitko NP, Grosser MR, Khatri D, Lance TR, Richardson AR. Expanded Glucose Import Capability Affords *Staphylococcus aureus* Optimized Glycolytic Flux during Infection. mBio. 2016;7(3). Epub 20160621. doi: 10.1128/mBio.00296-16. PubMed PMID: 27329749; PubMed Central PMCID: PMCPMC4916373.
