# Supplementary material for: The glutathione import system satisfies the Staphylococcus aureus nutrient sulfur requirement and promotes interspecies competition
Source: PLoS Genet. 2023 Jul 7;19(7):e1010834. doi: 10.1371/journal.pgen.1010834 (PMC10355420; doi:10.1371/journal.pgen.1010834)
Supplement: S2 Table — (DOCX) [file pgen.1010834.s003.docx]

| **S2 Table Primers used in this study.** | | |
| --- | --- | --- |
| **name** | **sequence 5’-3’** | **description** |
| pET28b::*ggt* F | AAGAAGGAGATATACCATGGTCATTAACTTAAATGACAAAC | amplify *ggt* ORF without stop codon to clone into pET28b |
| pET28b::*ggt* R | GATGATGGCTGCTGCTGCCCATGTCTTGTGATACTATCTCGAT | amplify *ggt* ORF without stop codon to clone into pET28b |
| pKOR1-mcs ∆*gis* upstream F | CTGCTAGCTAGCTAGAGATATCAAACGATAAAAAATATACAAATAAAAATCTAATTGTAG | amplify 1 kB upstream of SAUSA300_0201 to clone into pKOR1-mcs |
| pKOR1 ∆*gis* upstream R | AGCGTATAAAAAGTCATGCGTTGTGCAAC | amplify 1 kB upstream of SAUSA300_0201 to clone into pKOR1-mcs |
| pKOR1-mcs ∆gis downstream F | CGCATGACTTTTTATACGCTTGATATGAAGTTTG | amplify 1 kB downstream of SASUA300_0204 to clone into pKOR1-mcs |
| pKOR1-mcs ∆gis downstream R | CGG AAC CGG TAC CAA TGG ATA TCT ATG TTT TTG GCA ATG AAG TG | amplify 1 kB downstream of SAUSA300_0204 to clone into pKOR1-mcs |
| ∆*gis* conf. F | GACTAAGCTAAGTTGACACAC | confirmation of *gis* deletion |
| ∆*gis* conf. R | CATCCAAATCATCTATTAAAATCC | confirmation of *gis* deletion |
| pET28b::*gisA* F | ACTTTAAGAAGGAGATATACATGTCAAATTTATTAGAAGTCAAC | amplify *gisA* ORF without stop codon to clone into pET28b |
| pET28b::*gisA* R | AGTGGTGGTGGTGGTGGTGCGATTTAGCAATAACTGCTAC | amplify *gisA* ORF without stop codon to clone into pET28b |
| pOS1 P*_lgt_*::*ggt* F | ACAATTGAGGTGAACATATGGTCATTAACTTAAATGACAAAC | amplify *ggt* ORF to clone into pOS1 P*_lgt_* |
| pOS1 P*_lgt_*::*ggt* R | CTACCCCCTTGTTTGGATCCCTATCTTGTGATACTATCTC | amplify *ggt* ORF to clone into pOS1 P*_lgt_* reverse primer |
| pOS1 P*_lgt_*::*ggt*-his F | AAATACAATTGAGGTGAACATATGGTCATTAACTTAAATGACAAACAG | amplify *ggt* with his tag from pET28B::*ggt* to clone into pOS1 P*_lgt_* |
| pOS1 P*_lgt_*::*ggt*-his R | AGCTTGGCTGCAGGTCGACGGATCC  TCAGTGGTGGTGGTGGTG | amplify *ggt* with his tag from pET28B::*ggt* to clone into pOS1 P*_lgt_* |
