# Supplementary material for: The glutathione import system satisfies the Staphylococcus aureus nutrient sulfur requirement and promotes interspecies competition
Source: PLoS Genet. 2023 Jul 7;19(7):e1010834. doi: 10.1371/journal.pgen.1010834 (PMC10355420; doi:10.1371/journal.pgen.1010834)
Supplement: S1 Fig — (DOCX) [file pgen.1010834.s004.docx]

**S1 Fig.**

**
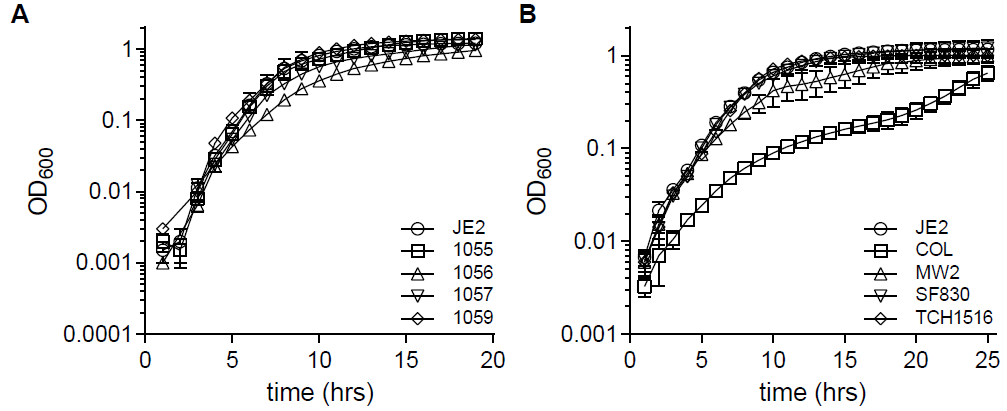
**

**S1 Fig. GSSG supplementation as the sole source of nutrient sulfur stimulates proliferation of *S. aureus*.** Laboratory derived JE2 and clinical isolates (**A**) or other laboratory strains (**B**) were cultured in medium containing 25 µM GSSG. The mean OD_600_ of three independent trials is depicted and error bars represent ± 1 standard error of the mean.
