# Supplementary material for: The glutathione import system satisfies the Staphylococcus aureus nutrient sulfur requirement and promotes interspecies competition
Source: PLoS Genet. 2023 Jul 7;19(7):e1010834. doi: 10.1371/journal.pgen.1010834 (PMC10355420; doi:10.1371/journal.pgen.1010834)
Supplement: S2 Fig — (DOCX) [file pgen.1010834.s005.docx]

**S2 Fig**


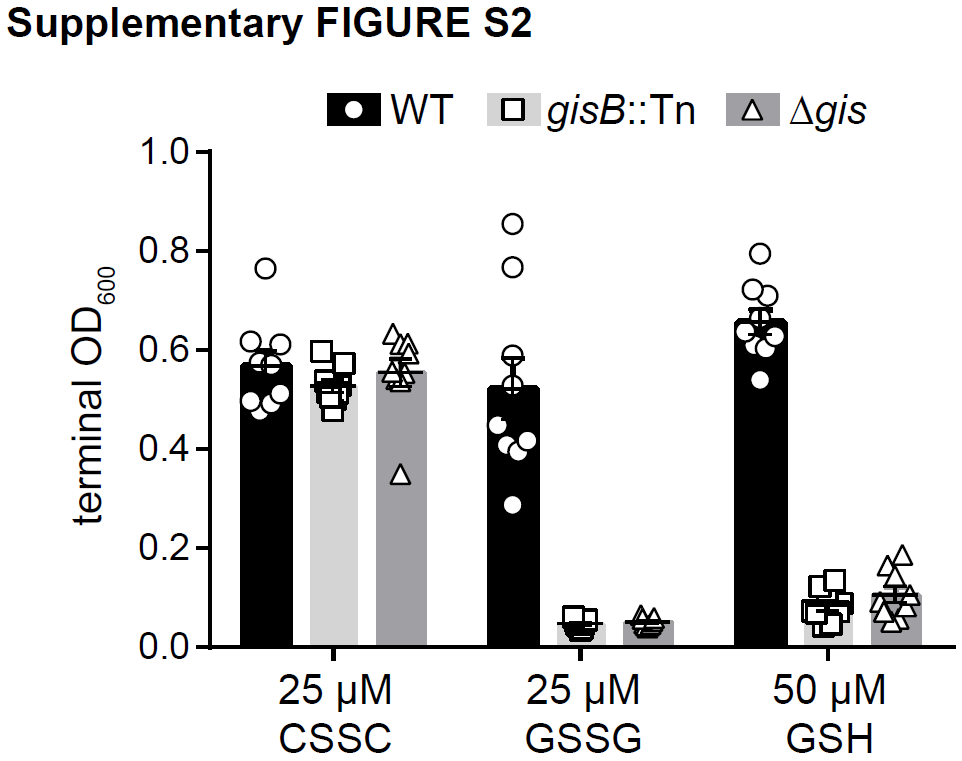


**S2 Fig. GisABCD-Ggt promotes anaerobic proliferation in PN medium supplemented with GSSG or GSH.** WT, *gisB*::Tn, and Δ*gisABCD-ggt* (Δ*gis*) were cultured in chemically defined PN medium in the presence of the alternative terminal electron acceptor sodium nitrate (100 mM) and the indicated sulfur sources. Sulfur source stock solutions were prepared anaerobically to maintain respective reduced or oxidized states. Each point represents the mean terminal OD_600_ after 24 h of growth derived from a technical triplicate (1 trial) and each bar represents the mean of nine independent trials. Error bars represent ± 1 standard error of the mean.
