# Supplementary material for: The glutathione import system satisfies the Staphylococcus aureus nutrient sulfur requirement and promotes interspecies competition
Source: PLoS Genet. 2023 Jul 7;19(7):e1010834. doi: 10.1371/journal.pgen.1010834 (PMC10355420; doi:10.1371/journal.pgen.1010834)
Supplement: S3 Fig — (DOCX) [file pgen.1010834.s006.docx]

**S3 Fig**


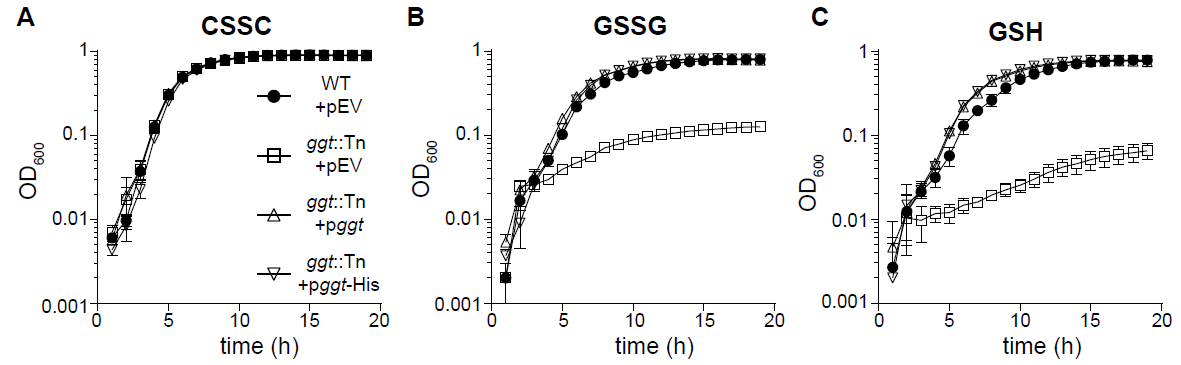


**S3 Fig. Ectopic expression of native or His-tagged Ggt complements *ggt* mutant proliferation in medium supplemented with reduced or oxidized GSH.** WT or *ggt*::Tn mutant strains harboring an empty pOS1 vector (pEV), pOS1 encoding *ggt* under the control of the constitutive promoter P*_lgt_* (p*ggt*), or a pOS1 vector encoding His-tagged *ggt* under the control of P*_lgt_* (p*ggt*-His) cultured in medium supplemented with 25 μM CSSC (**A**), 25 μM GSSG (**B**), or 50 μM GSH (**C**). Presented is the mean of at least three independent trials and error bars represent ± 1 standard error of the mean.
