# Supplementary material for: The glutathione import system satisfies the Staphylococcus aureus nutrient sulfur requirement and promotes interspecies competition
Source: PLoS Genet. 2023 Jul 7;19(7):e1010834. doi: 10.1371/journal.pgen.1010834 (PMC10355420; doi:10.1371/journal.pgen.1010834)
Supplement: S4 Fig — (DOCX) [file pgen.1010834.s007.docx]

| **S4 Fig**    **S4 Fig. Domain architectures and secondary structure predictions for the S. aureus GisABCD-Ggt system.** Domains were predicted with InterProScan (using MolEvolvR) [1-3], specifically using profile databases Pfam, ProSiteProfiles, and prediction algorithms, Phobius and TMHMM for the query proteins encoded by gisABCD-ggt: GisA (ABD21741.1), GisB (ABD21022.1) GisC (ABD20640.1), GisD (ABD22752.1), and Ggt (ABD22038.1). |
| --- |
| **Supporting References**  1. Burke JT, Chen SZ, Sosinski LM, Johnston JB, Ravi J. MolEvolvR: A web-app for characterizing proteins using molecular evolution and phylogeny. 2022.  2. Blum M, Chang HY, Chuguransky S, Grego T, Kandasaamy S, Mitchell A, et al. The InterPro protein families and domains database: 20 years on. Nucleic Acids Res. 2021;49(D1):D344-D54. doi: 10.1093/nar/gkaa977. PubMed PMID: 33156333; PubMed Central PMCID: PMCPMC7778928.  3. Quevillon E, Silventoinen V, Pillai S, Harte N, Mulder N, Apweiler R, et al. InterProScan: protein domains identifier. Nucleic Acids Res. 2005;33(Web Server issue):W116-20. doi: 10.1093/nar/gki442. PubMed PMID: 15980438; PubMed Central PMCID: PMCPMC1160203. |
