# Supplementary material for: The glutathione import system satisfies the Staphylococcus aureus nutrient sulfur requirement and promotes interspecies competition
Source: PLoS Genet. 2023 Jul 7;19(7):e1010834. doi: 10.1371/journal.pgen.1010834 (PMC10355420; doi:10.1371/journal.pgen.1010834)
Supplement: S5 Fig — (DOCX) [file pgen.1010834.s008.docx]

| **S5 Fig**  ****  **A**  ****  **C**  **B**  **S5 Fig. GisA encodes ATPase domain signatures and demonstrates ATP hydrolysis activity. A.** GisA contains two Walker A motifs, two Walker B motifs, and two ABC Signature motifs. Numbers correspond to the codon position of the first amino acid in the motif. Domain structure illustration was created with BioRender. **B.** Recombinant His-tagged GisA was expressed and purified from a modified E. coli NEB 3016 expression strain. Indicated samples and fractions were collected during expression and subsequent purification. Samples were loaded onto 12% SDS-PAGE and stained with Coomassie blue. Lanes: molecular weight ladder (M); 1: uninduced whole cell lysate (WCL); 2: WCL 4 h post IPTG induction; 3: pre-column lysate; 4: Ni-NTA flow-through; 5: and 6: 20 mM imidazole elution; 7: 100 mM imidazole elution; 8: and 9: 400 mM imidazole elution. GisA is predicted to be 59 kDa. **C.** Time course analysis of ATP hydrolysis activity of GisA incubated at 37°C for 1 h with 400 μM ATP. Samples were taken at the indicated time points and inorganic phosphate (P_i_) concentrations were determined using the malachite green assay. Presented is the mean of nine independent trials and error bars represent ± 1 standard error of the mean. Each trial used a new purification of GisA. |
| --- |
