# Supplementary material for: The glutathione import system satisfies the Staphylococcus aureus nutrient sulfur requirement and promotes interspecies competition
Source: PLoS Genet. 2023 Jul 7;19(7):e1010834. doi: 10.1371/journal.pgen.1010834 (PMC10355420; doi:10.1371/journal.pgen.1010834)
Supplement: S6 Fig — (DOCX) [file pgen.1010834.s009.docx]

| **S6 Fig**    **S6 Fig. Heterologous expression and purification of *S. aureus* Ggt from *Escherichia. coli*.** Recombinant His-tagged Ggt was expressed and purified from a modified *E. coli* NEB 3016 expression strain. Indicated samples and fractions were collected during expression and subsequent purification. Samples were loaded onto 12% SDS-PAGE and strained with Coomassie blue. Lanes: molecular weight ladder (M); 1. uninduced whole cell lysate (WCL); 2. WCL 4 h post IPTG induction; 3. pre-column lysate; 4. Ni-NTA column flow-through; 5. 20 mM imidazole wash; 6. 50 mM imidazole elution; 7. 100 mM imidazole elution; fractions 8. and 9. consecutive 400 mM imidazole elutions. |
| --- |
