# Supplementary material for: The glutathione import system satisfies the Staphylococcus aureus nutrient sulfur requirement and promotes interspecies competition
Source: PLoS Genet. 2023 Jul 7;19(7):e1010834. doi: 10.1371/journal.pgen.1010834 (PMC10355420; doi:10.1371/journal.pgen.1010834)
Supplement: S7 Fig — (DOCX) [file pgen.1010834.s010.docx]

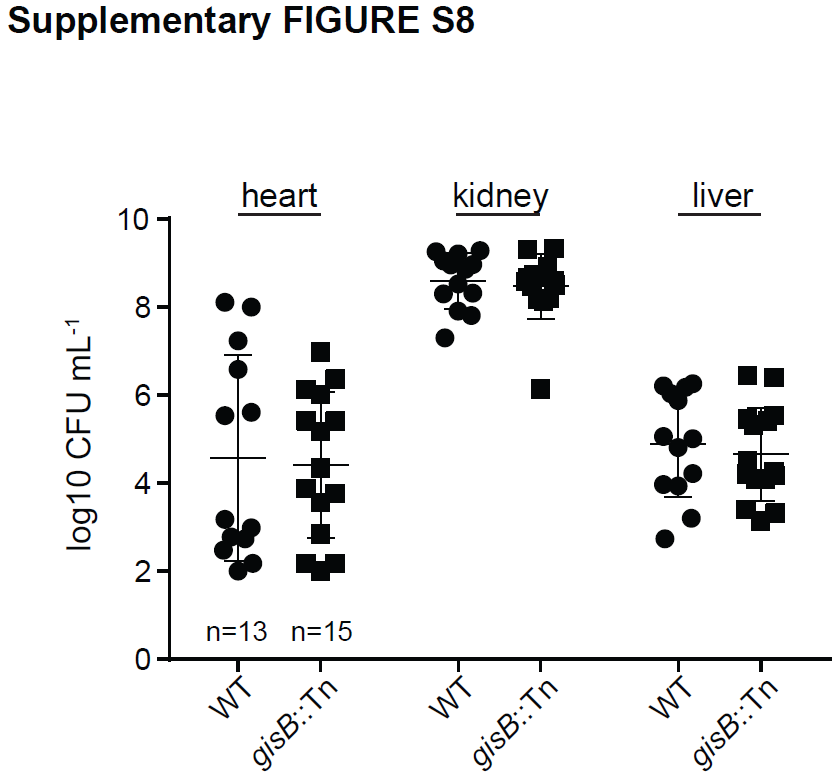
**S7 Fig**

**S7 Fig. Virulence of a gisB::Tn mutant strain mimics wild type.** Bacterial burdens within indicated organs of C57BL/6J mice were enumerated after 96 h of systemic infection with either WT (circles) or gisB::Tn (squares). Bacterial burdens are presented as log_10_ CFUs mL^-1^ for liver, combined kidneys, and heart. The mean and standard deviation are shown. Each symbol represents one animal.
