# Supplementary material for: The glutathione import system satisfies the Staphylococcus aureus nutrient sulfur requirement and promotes interspecies competition
Source: PLoS Genet. 2023 Jul 7;19(7):e1010834. doi: 10.1371/journal.pgen.1010834 (PMC10355420; doi:10.1371/journal.pgen.1010834)
Supplement: S8 Fig — (DOCX) [file pgen.1010834.s011.docx]

**S8 Fig**

***
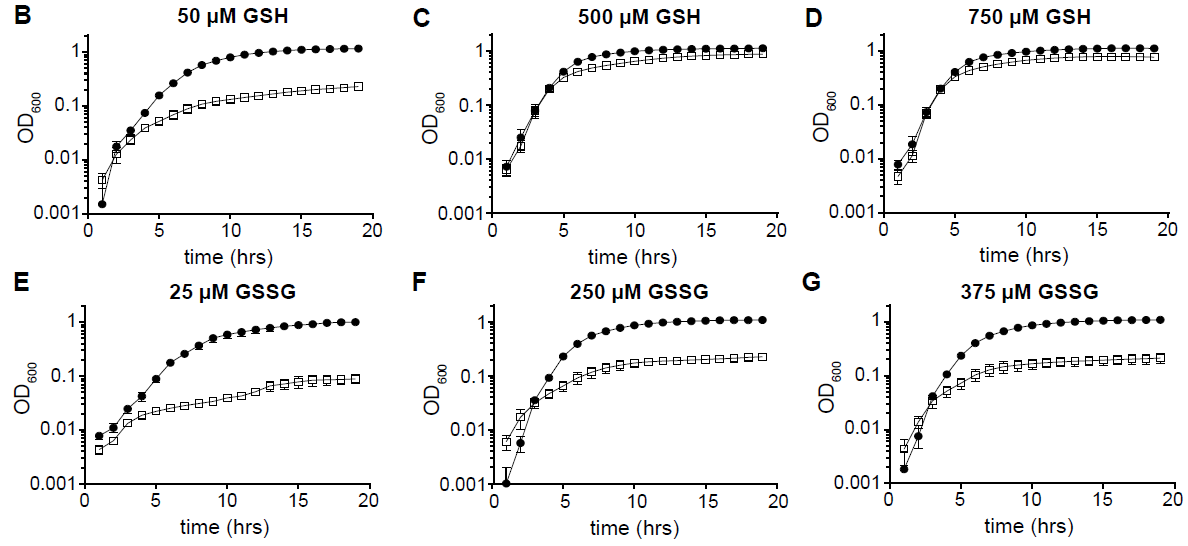
*
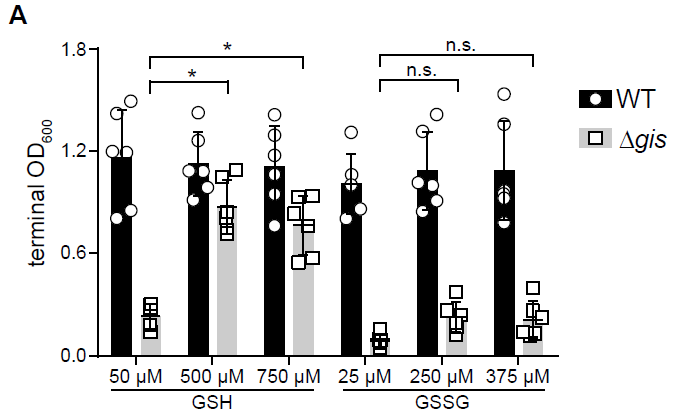
**

**S8 Fig. S. aureus acquires GSH independent of GisABCD-Ggt in physiologically relevant concentrations of GSH**. **A.** WT and ∆gisABCD-ggt (∆gis) were cultured in medium supplemented with 50 µM GSH, 500 µM GSH, 750 µM GSH, 25 µM GSSG, 250 µM GSSG, or 375 µM GSSG. Bars depict the mean OD_600_ after 19 hrs of growth. Dots represent the terminal OD_600_ from each individual trial. Error bars represent the standard deviation. ***** denotes P-value <0.05 by one-way ANOVA with Tukey’s multiple comparison correction. **B-G**. Growth curves for WT or ∆gisABCD-ggt (∆gis) cultured in increasing concentrations of GSH or GSSG. The mean of at least three independent trials is presented and error bars represent ± 1 standard error of the mean. ***** denotes P-value <0.05 by one-way ANOVA with Tukey’s multiple comparison correction. The mean of at least three independent trials is presented. Error bars represent ± 1 standard error of the mean.
