# Supplementary material for: The glutathione import system satisfies the Staphylococcus aureus nutrient sulfur requirement and promotes interspecies competition
Source: PLoS Genet. 2023 Jul 7;19(7):e1010834. doi: 10.1371/journal.pgen.1010834 (PMC10355420; doi:10.1371/journal.pgen.1010834)
Supplement: S9 Fig — (DOCX) [file pgen.1010834.s012.docx]

**S9 Fig**

**S9 Fig. Conservation of Ggt and GisABCD across Firmicutes.** Percent similarity of GisABCD-Ggt homologues with respect to the S. aureus proteins. Results were limited to Firmicutes encoding proteins that harbor an annotated glutamyl transpeptidase domain based on MolEvolvR analysis and the presence of the Pfam Ggt domain. S. aureus GisABCD was subsequently queried within Ggt-encoding genomes. The dendrogram on the left uses hierarchical clustering to group genomes with similar GisABCD-Ggt co-occurrence patterns.
