# Supplementary material for: The glutathione import system satisfies the Staphylococcus aureus nutrient sulfur requirement and promotes interspecies competition
Source: PLoS Genet. 2023 Jul 7;19(7):e1010834. doi: 10.1371/journal.pgen.1010834 (PMC10355420; doi:10.1371/journal.pgen.1010834)
Supplement: S10 Fig — (DOCX) [file pgen.1010834.s013.docx]

**S10 Fig**

**
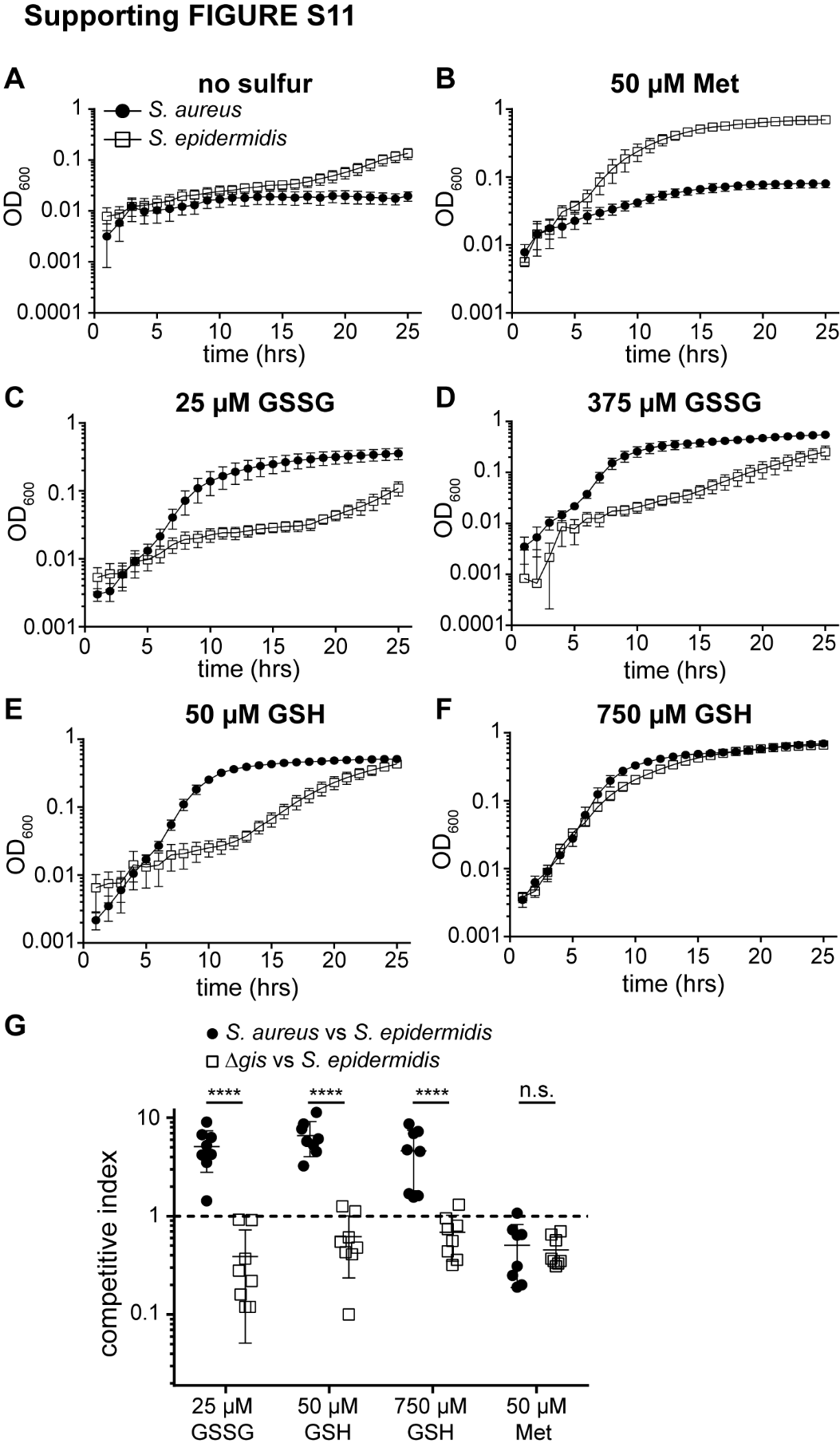
**

**S10 Fig. S. epidermidis and S. aureus nutrient sulfur source utilization is distinct and promotes interspecies competition. A-F.** S. aureus and S. epidermidis were cultured in PN_mod_ containing the indicated source of nutrient sulfur. The mean OD_600_ of at least three independent trials and error bars depicting ± 1 standard error of the mean are presented. **G.** In vitro competition between S. epidermidis and S. aureus or S. aureus ∆gisABCD-ggt (∆gis) in PN_mod_ containing the indicted sources of nutrient sulfur. The competitive index of each trial is presented. The mean and standard deviation are presented. **** indicates P-value <0.0001 as determined by one-way ANOVA with a Sidak multiple test correction.
