# Supplementary material for: The glutathione import system satisfies the Staphylococcus aureus nutrient sulfur requirement and promotes interspecies competition
Source: PLoS Genet. 2023 Jul 7;19(7):e1010834. doi: 10.1371/journal.pgen.1010834 (PMC10355420; doi:10.1371/journal.pgen.1010834)
Supplement: Attachment — Submitted filename: Lensmire JM.docx [file pgen.1010834.s015.docx]

The manuscript entitled “The glutathione import system satisfies the *Staphylococcus aureus* nutrient sulfur requirement and promotes interspecies competition” by Lensmire, JM *et al.* describes a locus that encodes an ABC transporter for GSH and GSSG as well as the first enzyme in the breakdown of GSH, Ggt. The authors demonstrate substrate specificity and calculate Michaelis-Menten parameters for each substrate. The authors also demonstrate the limited distribution of similar systems among a select clade of Firmicutes. While the Gis transport system does not seem to directly contribute to overall virulence in *S. aureus*, it does seem to promote competition between species that lack such transporters. This, however, is difficult to put into real world context as *S. aureus* would most likely encounter species that lack Gis while colonizing the skin surface, an environment not known to be replete with GSH. Finally, the authors localize Ggt to the cytosol, a rare compartmentalization for such enzymes that are usually found extracellularly or in the periplasm. A few minor additions might improve the impact of the manuscript:

1. Do the authors know anything about the enzyme that separates Gly from Cys after the reaction with Ggt or can *S. aureus* use the sulfur directly from the Cys-Gly dipeptide?
2. How is the Gis system regulated transcriptionally? Is anything known about what induces the system?
3. Instead of screening for other sulfur sources that could be transported by Gis, are other tri-peptides transported by the system? It may not be selective for solely sulfur containing peptides.
4. With the inframe deletion mutant in the Gis locus, a screen of the Tn library should be able to easily identify the second low affinity GSH transporter. This would add dramatically to the impact of the story.
