# Supplementary material for: The glutathione import system satisfies the Staphylococcus aureus nutrient sulfur requirement and promotes interspecies competition
Source: PLoS Genet. 2023 Jul 7;19(7):e1010834. doi: 10.1371/journal.pgen.1010834 (PMC10355420; doi:10.1371/journal.pgen.1010834)
Supplement: Attachment — Submitted filename: Lensmire et al response to reviewers.docx [file pgen.1010834.s016.docx]

**Response to Reviewers**

Lensmire *et al.* - PGENETICS-D-23-00230

**Minor Editor comments***:*

*A few minor points:*

*Line 52: please add a comma after "operon"*

*Line 94: please add "it" after "but"*

*Line 296: please delete "compared to" at the end of the sentence*

We thank the Editors for catching these typographical errors and made the indicated changes (currently line 52, line 95, and line 311, respectively).

‘***Please also add a little more detail about how the transposon screening was done to the Materials and Methods*.**’

This request for additional information adds to the clarity of our work and we thank the Editors for this suggestion. The new information is presented in the **Materials and Methods** section titled ‘*Isolation of GSSG-proliferation impaired transposon mutants*’ (Lines 475-501) and includes a more comprehensive description of our primary and secondary screening procedure.

**Review #1**

***1) Please demonstrate GSH/GSSG transport by the Gis system. Otherwise, renaming the genes and ascribing function is premature, and the predicted factors should be labeled as ‘putative’****.*

We thank the Reviewer for urging us to demonstrate GSSG transport by the GisABCD system. The experiment has been conducted and the results are included in the manuscript (**Fig. 3**). Briefly, we used a tandem mass spec. approach to detect GSSG in WT and the *ggt* transposon mutant, but not in a strain harboring and inframe *gisABCD-ggt* deletion (Δ*gis*). Each strain was cultured in PN media supplemented with cystine (CSSC) as the sulfur source to mid-exponential phase. The cells were then washed, incubated in the presence or absence of 25 µM GSSG for 5 min, and collected for subsequent analysis. We were not able to detect GSSG across the three strains without GSSG supplementation. GSSG was also not detected in the Δ*gis* mutant after a 5 min exposure to 25 µM GSSG; however, GSSG was present in both WT and the *ggt* mutant, both of which encode the *gisACBD* transporter. These data increase the rigor of work by demonstrating that cells expressing *gisABCD* are capable of acquiring GSSG. Together with the growth phenotypes and bioinformatic analyses, the results provide additional support for the conclusion that GisABCD functions as an importer.

***2) Please expand on the Gis/Ggt link that is suggested by Fig. S7. Are there thoughts on potential protein-protein interactions between Ggt and the transporter, with imported GSH/GSSG immediately cleaved? Along these lines, please include in the main text this or a similar cartoon of the importer and Ggt, with proposed mechanisms that fuel sulfur-based growth physiology.***

Our contention is that it is premature to speculate on direct interactions between the Gis proteins as our results do not provide evidence of such. Nonetheless, this is an interesting idea that drives an area of research in our lab.

We moved the illustration of the model from Supplemental Fig. S7 to Figure 4F in the main text. We thank the reviewer for this helpful suggestion.

***3) Please reconsider the competition data because it seems relatively contrived, with the outcomes dictated by how species individually grow, not their interactions. While I don’t mean to propose a gain-of-function experiment, are the authors suggesting that acquisition of gis genes by S. epidermidis would enable the species to grow on GSH?***

We agree with Reviewer #1 that results competing WT *S. aureus* against WT *S. epidermidis* are consistent with expectations based on the sulfur source growth analyses presented in Fig. S10 (formerly Fig. S11). However, the notable result in these experiments is the significantly decreased capacity of Δ*gis* *S. aureus* to compete with WT *S. epidermidis* when either GSSG or GSH are supplied as the sole source of sulfur. This finding supports the conclusion that Gis-Ggt promotes accelerated *S. aureus* proliferation over *S. epidermidis*. The implication of this result is that Gis-Ggt promotes colonization of *S. aureus* over *S. epidermidis* in GSH- or GSSG-supplemented environments. Though, currently there is no direct evidence that such an environment exists in the context of mammalian tissues. We are careful not to allude to a direct interaction between the two strains, because, as Reviewer #1 rightfully points out, this is a limitation of the experiment. For these reasons we refrain from stating that a specific interaction is taking place between the microorganisms.

Our results support the hypothesis that a *S. epidermidis* strain expressing the Gis-Ggt system will compete with WT *S. aureus* in the presence of GSH or GSSG resulting in a competitive index near 1. Though, we have not tested that directly due to the gain-of-function concerns.

***4) In Fig. 4, please incorporate a simple 16S tree to organize the species (along with an appropriate outgroup).***

We are grateful for Reviewer #1’s insightful comment and have accordingly incorporated the 16S tree into Fig. 5B (which was formerly Fig. 4).

***5) Lines 80-1: This statement seems inherently incorrect. There are many non-pathogenic microbes for which sulfur acquisition mechanisms have been explored, e.g. E. coli, various symbiotic microbes, and certainly marine organisms.***

We thank the reviewer for pointing out this overstatement and lack of clarity. To address this, we replaced the sentence with the following (Lines 79-82):

‘*While strategies employed by bacteria to acquire nutrient transition metals such as iron have been an intense area of research, studies defining mechanisms of nutrient sulfur procurement from host environment, are garnering heightened interest* [1-6].’

***6) To what extent does overexpression of Ggt affect growth of the gis mutants in GSH?***

We appreciate the question and performed the experiment. The results are presented below. Overexpression of Ggt is driven by increased plasmid copy number and a constitutive promoter called P*_lgt_* (the vector is referred to as p*ggt* in the figure legend, EV is the empty vector lacking *ggt*). Our conclusion is that overexpression of Ggt in the Δ*gis* mutant (denoted as Δ*gis*-*ggt* in the legend) does not compensate for loss of the ABC transporter subunits. We consider this to be a negative result and for these reasons did not include the data in the revised manuscript.

***7) For the species that lack Gis, can gene synteny reveal whether gis undergoes horizontal gene transfer? Do the genes surrounding gis/ggt in S. aureus neighbor each other in S. epidermidis?***

We were also hoping that the bioinformatic analyses would help deduce the origin of Gis-Ggt. However, no obvious elements of horizontal gene transfer (i.e. transposons, insertion elements, prophages etc.) are present within or near the loci of interest in *S. aureus* or *S. epidermidis*. We present an illustration of the synteny between the *S. aureus* and *S. epidermidis* loci below but have not included them in the manuscript due to the lack of a conclusive results.

Method: To evaluate gene neighborhood, protein sequences from Ggt (SAUSA300_0204) and GisB (SAUSA300_0201) were used as input in webFlaGs (Saha et al., 2021), selecting default parameters with 7 flanking genes.

Reference: Saha CK, Pires RS, Brolin H, Delannoy M, Atkinson GC. FlaGs and webFlaGs: discovering novel biology through the analysis of gene neighbourhood conservation. Bioinformatics. 2021;37: 1312–1314. doi:10.1093/BIOINFORMATICS/BTAA788

We extended our analysis beyond the handful of genes presented here, but also failed to identify obvious genes related to horizontal gene transfer. We thank the reviewer for this perceptive question.

**Reviewer #2:**

***1. I am intrigued by the growth curve in PN medium lacking sulfur in Figure 1A. Growth in this medium has a similar growth rate as PN medium containing GSH-GSSG for a short period of time but then abruptly levels off and does not have further exponential growth. What is the source of the sulfur facilitating early exponential growth? Is it contamination of sulfur in the water or potentially methionine?***

This is an astute observation. Based on comparing *S. aureus* proliferation in media containing methionine (Figs. 1, 2, S3B, S3B, S8B, S8E, S8F, and S8G) to that without methionine (Fig. S10A) we surmise that methionine is playing a role in this slight growth promotion. The two most likely possibilities are that (i) *S. aureus* inefficiently utilizes methionine as a source of nutrient sulfur or (ii) an impurity in our stock methionine. Genetics support the latter, as *S. aureus* does not encode a complete, conical methionine synthesis pathway. We purchased the methionine from Fischer Scientific (item number BP388-100) and recently discovered this version has been discontinued. The product specifications state that other amino acids were ‘*chromatographically not detectable*’. Overall, the purity of the reagent is stated to be 98.5%-100%. We have also tested whether fratricide was a possible mechanism, but decreasing the inoculum does not change the growth kinetics (data not shown). Based on these findings, we believe the phenotype warrants additional investigation, but our contention is that it does not take away from the core conclusions of this work.

***2. Although not necessarily essential for this manuscript, it would add significant rigor if a transport assay would be performed with 3H GSH-GSSG to document that GisABCD is a transporter. Further, is it possible to construct a gisABCD-ggt/tcyP/tcyABC mutant? Or is that mutant not viable? This would be interesting and a good fit for this manuscript regarding the animal model of infection.***

We thank the reviewer for suggesting that we pursue increased rigor of our work by tracking GSSG uptake. We performed the experiment, and the results are described in the response to Reviewer #1’s comment #1.

We have attempted to construct the *gis-ggt tcyP tcyA* triple mutant, but unforeseen technical issues have plagued the validation of the resulting mutant. We are currently addressing these issues but agree with the reviewer that this experiment could shed light on the importance and potential redundancy between *S. aureus* acquisition of cysteine, GSH, and GSSG as sources of nutrient sulfur during infection.

***3. Methicillin-susceptible throughout instead of methicillin-sensitive***

We thank the reviewer for recognizing this mistake and made the appropriate corrections (Lines: 137 and 506)

***4. Streptococcus pneumoniae throughout instead of Streptococcus pneumonia***

We are grateful that the reviewer caught this typographical error and have made the corrections (Line: 372).

**Reviewer #3**

***1. Do the authors know anything about the enzyme that separates Gly from Cys after the reaction with Ggt or can S. aureus use the sulfur directly from the Cys-Gly dipeptide?***

This is an interesting question, but we did not isolate any other peptidase mutants in our primary screen. We have also embarked on directed approaches by knocking out known peptidases and none of the single mutants have resulted in a phenotype, potentially due to redundancy. This is the case in *E. coli* where multiple peptidases are capable of cleaving Cys-Gly

We have data that supports the conclusion that *S. aureus* is capable of using Cys-Gly as a source of nutrient sulfur and we have identified the transporter. Importantly for this work, Gis-Ggt is not involved; therefore, our contention is that these results warrant an independent publication.

***2. How is the Gis system regulated transcriptionally? Is anything known about what induces the system?***

Interestingly, Soutourina and colleagues reported that *gisA*, but not *gisB-ggt*, was found to be elevated mutant *S. aureus* genetically inactivated for the major sulfur response regulator CymR compared to WT (*Mol. Micro.*, 2009, PMID: 19508281). We are currently preparing a manuscript that describes CymR regulation of *gisABCD-ggt* further in work that defines the *S. aureus* CymR-dependent and -independent sulfur starvation response. We appreciate this perceptive thought from Reviewer #3 but believe that the regulation of the system is beyond the scope of this publication.

***3. Instead of screening for other sulfur sources that could be transported by Gis, are other tri-peptides transported by the system? It may not be selective for solely sulfur containing peptides.***

This is an excellent question and something we are currently pursuing. Our preliminary results suggest that *S. aureus* is capable of utilizing *S*-nitrosoglutathione (GSNO) as a sulfur source. However, data defining the role of Gis-Ggt in this process are inconclusive at this point. Our perspective is that these results are too preliminary and extend beyond the initial identification and characterization of the system.

***4. With the inframe deletion mutant in the Gis locus, a screen of the Tn library should be able to easily identify the second low affinity GSH transporter. This would add dramatically to the impact of the story.***

We thank the reviewer for the idea and have made several attempts to identify the additional GSH transporter using TnSeq. Thus far technical issues have frustratingly made completing this experiment difficult to accomplish. It’s a great thought nonetheless.
